# Diffusion-weighted MR image analysis based on gamma distribution model for differentiating benign and malignant brain tumors

**DOI:** 10.1097/MD.0000000000039593

**Published:** 2024-09-06

**Authors:** Zeinab Soleimani, Masih Saboori, Iraj Abedi, Maziar Irannejad, Saeid Khanbabapour

**Affiliations:** a Department of Medical Physics, School of Medicine, Isfahan University of Medical Sciences, Isfahan, Iran; b Department of Neurosurgery, School of Medicine, Isfahan University of Medical Sciences, Isfahan, Iran; c Department of Electrical Engineering, School of Electrical Engineering, Islamic Azad University Najafabad Branch, Najafabad, Iran; d Department of Imaging, Asgarieh Hospital, Isfahan, Iran.

**Keywords:** apparent diffusion coefficient, gamma diffusion model, glioma, magnetic resonance imaging, meningioma

## Abstract

**Background:**

Considering the invasiveness of the biopsy method, we attempted to evaluate the ability of the gamma distribution model using magnetic resonance imaging images to stage and grade benign and malignant brain tumors.

**Methods:**

A total of 42 patients with malignant brain tumors (including glioma, lymphoma, and choroid plexus papilloma) and 24 patients with benign brain tumors (meningioma) underwent diffusion-weighted imaging using five *b*-values ranging from 0 to 2000 s/mm^2^ with a 1.5 T scanner. The gamma distribution model is expected to demonstrate the probability of water molecule distribution based on the apparent diffusion coefficient. For all tumors, the apparent diffusion coefficient, shape parameter (*κ*), and scale parameter (*θ*) were calculated for each *b*-value. In the staging step, the fractions (ƒ_1_, ƒ_2_, ƒ_3_) expected to reflect the intracellular, and extracellular diffusion and perfusion were investigated. Diffusion <1 × 10^‐4^ mm^2^/s (ƒ_1_), 1 × 10^‐4^ mm^2^/s < Diffusion > 3 × 10^‐4^ mm^2^/s (ƒ_2_), and Diffusion >3 × 10^‐4^ mm^2^/s (ƒ_3_); in the grading step, fractions were determined to check heavily restricted diffusion. Diffusion lower than 0.3 × 10^‐4^ mm^2^/s (ƒ_11_). Diffusion lower than 0.5 × 10^‐4^ mm^2^/s (ƒ_12_). Diffusion lower than 0.8 × 10^‐4^ mm^2^/s (ƒ_13_).

**Results:**

The findings were analyzed using nonparametric statistics and receiver operating characteristic curve diagnostic performance. Gamma model parameters (*κ*, ƒ_1_, ƒ_2_, ƒ_3_) showed a satisfactory difference in differentiating meningioma from glioma. For *b* value = 2000 s/mm^2^, ƒ_1_ had a better diagnostic performance than *κ* and apparent diffusion coefficient (sensitivity, 88%; specificity, 68%; *P* < .001). The best diagnostic performance was related to ƒ_3_ in *b* = 2000 s/mm^2^ (area under the curve = 0.891, sensitivity = 83%, specificity = 80%, *P* < .001). In the grading step, ƒ_12_ (area under the curve = 0.870, sensitivity = 92%, specificity = 72%, *P* < .001) had the best diagnostic performance in differentiating high-grade from low-grade gliomas with *b* = 2000 s/mm^2^.

**Conclusion:**

The findings of our study highlight the potential of using a gamma distribution model with diffusion-weighted imaging based on multiple *b*-values for grading and staging brain tumors. Its potential integration into routine clinical practice could advance neurooncology and improve patient outcomes through more accurate diagnosis and treatment planning.

## 1. Introduction

Definitive diagnosis of benign versus malignant brain tumors is typically performed through biopsy for histological confirmation.^[1]^ However, biopsies are generally not performed before surgery because of associated risks. Diffusion-weighted magnetic resonance imaging (MRI) (DWI) is a valuable tool for distinguishing between different brain tumors, reducing the need for invasive biopsies. DWI is a noninvasive technique that measures water diffusion in tissues and provides essential information about the tissue microstructures that play a crucial role in tumor grading.^[2]^

Various histological tumor types exhibit different cellularity’s, leading to variations in DWI intensity. Multiple mathematical models have been proposed to analyze DWI MR images. A commonly used model is the mono-exponential model, which calculates the apparent diffusion coefficient (ADC) based on the assumption of Gaussian distribution of diffusion displacement.^[3]^ Although this model has shown some utility in differentiating tumors, it has some inherent limitations. In reality, diffusion behavior in heterogeneous biological tissues cannot be accurately represented by a simple Gaussian distribution.

Several approaches have been proposed to address the limitations of the mono-exponential model and characterize non-mono-exponential diffusion behavior.^[4]^ In recent years, statistical models based on gamma distribution have been proven to be suitable for diffusion MRI analyses. The gamma distribution (GD) model is a two-parameter continuous probability distribution parameterized by the shape parameters kappa (κ) and scale parameter theta (θ). This model assumes that the diffusion coefficient (*D*) is continuously distributed within a voxel, thereby enabling the estimation of fractions representing different tissue types. Specifically, the area fractions for *D* < 1.0 × 10^−3^ mm^2^/s, *D* = 1.0 × 10^−3^ to 3.0 × 10^−3^ mm^2^/s, and *D* > 3.0 × 10^−3^ mm^2^/s are attributed to intracellular, extracellular extravascular, and intravascular spaces, respectively. By using these fractions, it is possible to estimate the histopathological conditions of neoplasms or organs.

The gamma model introduces the concept of area fractions for diffusion coefficients *D* < 1.0 × 10^−3^ mm^2^/s and *D* > 3.0 × 10^−3^ mm^2^/s as parameters representing restricted diffusion and perfusion, respectively. With the GD model’s continuous distribution of diffusion coefficients within the imaging voxel, the histological interpretation of diffusion data becomes feasible.^[5]^ Although the GD model has been successfully applied in assessing prostate cancers,^[6]^ breast cancers,^[5]^ and renal function,^[7]^ its application to brain tumors remains unexplored. Hence, the primary aim of this study was to investigate the potential of the GD model to differentiate between benign and malignant brain tumors. Our research comprises 2 main parts: first, we examine the diagnosis of benign and malignant tumors based on the probability density function (PDF) of the gamma model, and second, we investigate the classification of malignant tumors by altering their fractions.

Through this study, we strive to provide valuable insights into the potential application of the GD model in brain tumor analysis, potentially enhancing the diagnostic accuracy and reducing the need for invasive procedures in clinical practice.

## 2. Materials and methods

### 2.1. Patients

A total of 73 patients with brain tumors, referred for MRI examination between May 2021 and February 2023, participated in this fundamental and prospective study. The study protocol was approved by the Institutional Review Board of Isfahan University of Medical Sciences (number: 1400.641), and informed consent was obtained from all the patients. The inclusion criteria required confirmation of brain tumors by a neurologist, and patients were excluded if they had contraindications for MRI imaging, such as cardiac pacemakers, aneurysm clips, artificial heart valves, or body prostheses. Patients with foreign objects such as splinters or pleats were also excluded to prevent potential artifacts. Patients who had undergone prior surgery or biopsy were excluded from the study. Patients with low-quality diffusion MRI images were excluded. After MRI examination, the patients underwent surgery, and tissue samples were sent to the pathology laboratory for tumor staging and grading.

### 2.2. Image acquisition

MR images were obtained using a 1.5T MRI scanner (Aera, Siemens Medical Systems, Germany) equipped with an 8-channel head coil in Milad Hospital. The DWI sequence was acquired in the axial plane with 3 orthogonal diffusion gradient directions at 5 *b* value (0, 500, 1000, 1500, and 2000 s/mm^2^). The imaging parameters were as follows: repetition time = 3000 ms; echo time 102 ms; slice thickness, 5 mm; field of view 250 × 250 mm²; fat suppression, SPIR; water-fat shift/ BW, 9.2 pixels/23.6 Hz; BW in EPI-frequency direction. 1680.3 Hz; acquisition time, 1 minutes and 12 seconds; and matrix size, 256 × 256. Each *b*-value was calculated as the average of 3 measurements.

In addition to DWI, routine MRI protocols were performed for all patients, which included T1-weighted sequences with and without contrast, T2-weighted sequences in the axial, sagittal, and coronal planes, and fluid-attenuated inversion recovery sequence in the axial plane. *B*-values were selected according to previous studies^[8]^ and internal guidelines of our department for brain tumor patients.

### 2.3. Regions of interest placement

All MR images were meticulously analyzed by an experienced neuro-radiologist with 10 years of MRI expertise. The radiologist was blinded to the patients’ medical history and pathology results. To match the geometric information of the DWI images, the matrix sizes of the post-contrast T1-weighted images were adjusted using the Image J function.

**Figure 1.**
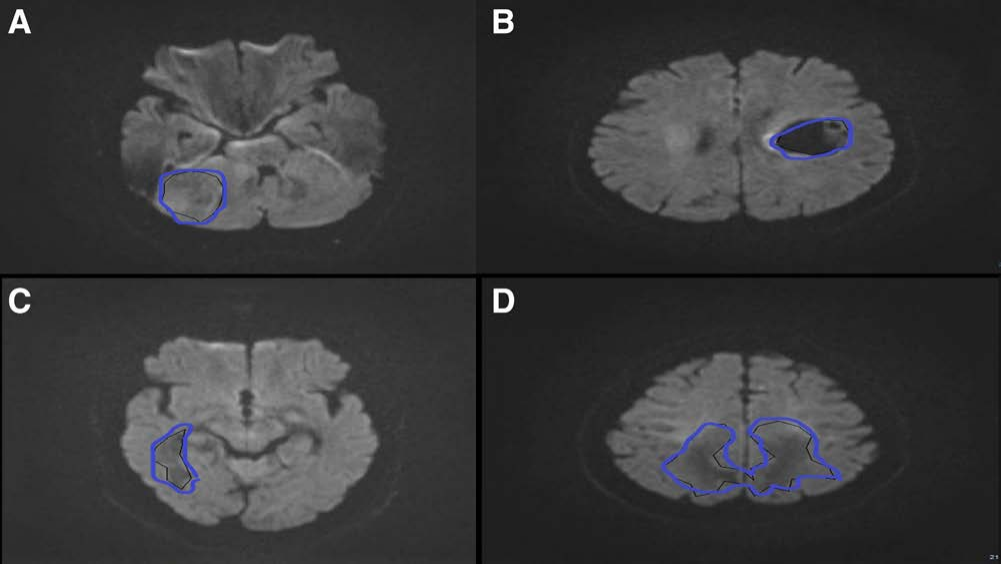
Region of interest (ROI) at *b*_value = 2000, (A) meningioma in a 51 years-old woman, (B) glioma grade I in a 46 years-old woman, (C) glioma grade II in a 27 years-old man, (D) glioma high-grade in a 58 years-old man.

Regions of interest (ROI) were defined to include the conspicuous core of the lesion, enclosing the limits of the viable hyperintense regions of the lesions and avoiding peritumoral edema, hemorrhage, and cystic lesions (Fig. 1).

**Figure 2.**
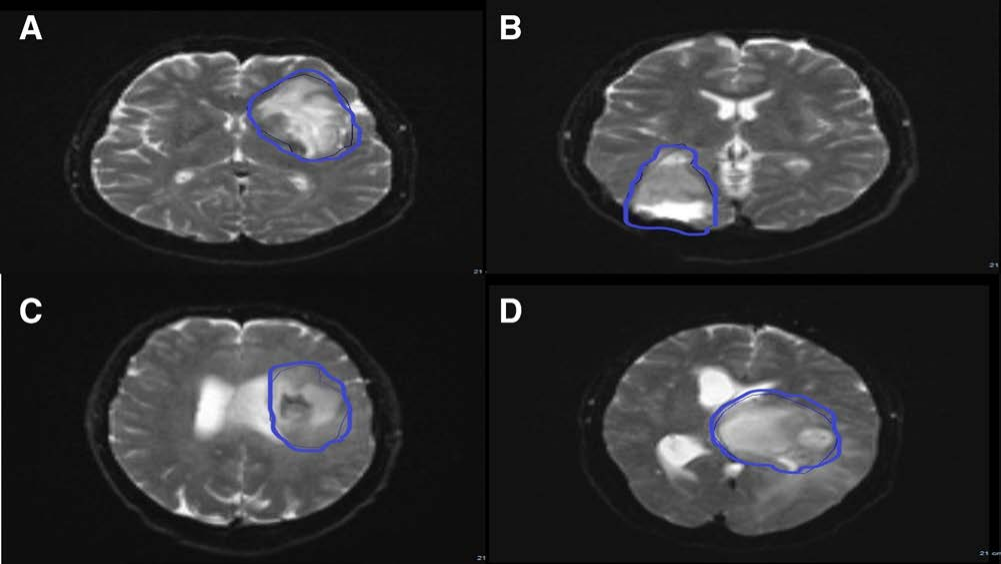
Region of interest (ROI). (A) Glioma grade II in 33 years old male. (B) Glioma grade IV in 45 years old female. (C) Glioma grade IV in 65 years old male. (D) Glioma grade III in 40 years old female.

For the grading step, ROIs were carefully placed to delineate the enhancing lesion on the single slice with the maximum area in T1w post-contrast, utilizing a 3D-slicer (v5.4.0 × 64). ROIs were excluded from areas with necrosis, cystic lesions, hemorrhage, or obvious artifacts (Fig. 2).

To ensure consistency, these ROIs were copied and pasted onto the images corresponding to the other b value. Fine manual adjustments were performed to address any locational mismatches caused by image distortion or patient motion, thereby ensuring accurate ROI placement. ROIs were strategically selected to encompass the core of the tumor while avoiding regions of edema, hemorrhage, or normal tissue. The mean signal intensity of each ROI was measured at the desired b value using in-house software (MATLAB-R2016b).

### 2.4. DWI imaging analysis

ADC maps were derived from the acquired diffusion-weighted images using MATLAB-R2016b, a software widely used for image analysis. ADC maps were generated by summing multi-*b*-value images. Subsequently, ROIs were drawn independently on the ADC maps and the ADC values for all slices were calculated using the following formula:

$$\begin{array}{l} ADC=-\frac{1}{b}\times ln\left(\frac{S_{DWI}}{S_{b=0}}\right)\;\;mm^{2}/s \end{array}$$ (1)

where *S*_DWI_ is the signal intensity of isotropic DWI, *S*_*b*=0_ is the signal intensity of *b* = 0, and ADC is the apparent diffusion coefficient.

The GD model is denoted by *P*(*D*) and is defined as follows:

$$\begin{array}{l} P(D)=\frac{1}{\;\Gamma \;(k)\theta^{k}}D^{K-1}\;exp^{\frac{-D}{\theta }} \end{array}$$ (2)

where:

- *P*(*D*) represents the PDF of the gamma distribution.
- *κ* is the shape parameter of the distribution.
- *θ* is the scale parameter of the distribution.
- Γ denotes the gamma function.
- *D* is the diffusion coefficient.

This statistical model enables us to estimate the fractions of tissue types based on specific ranges of diffusion coefficients, providing valuable information for distinguishing between benign and malignant brain tumors.

The following formula was used to calculate the parameters of the gamma model (θ/k) based on signal intensity:

$$\begin{array}{l} S\left(b\right)=S_{0}\frac{1}{{\left(1+\theta b\right)}^{k}} \end{array}$$ (3)

The study was conducted in 2 parts to investigate the potential of the gamma distribution model for staging and grading brain tumors.

### 2.5. Staging step

First, the diagnosis of benign and malignant tumors was explored based on the PDF of the gamma model. Diffusion-weighted MR images were analyzed using the gamma distribution model to identify 3 distinct areas in the function curve: diffusion lower than 1 × 10^‐4^ mm^2^/s (Frac < 1 × 10^‐4^ mm^2^/s): which is expected to represent small intracellular diffusion. Diffusion between 1 × 10^‐4^ mm^2^/s and 3 × 10^‐4^ mm^2^/s (1 × 10^‐4^ mm^2^/s < Frac > 3 × 10^‐4^ mm^2^/s): this region corresponds to the extracellular diffusion of the tissue. Diffusion higher than 3 × 10^‐4^ mm^2^/s (Frac > 3 × 10^‐4^ mm^2^/s): This region was expected to indicate the perfusion component.

### 2.6. Grading step

The second part of the study focused on grading malignant brain tumors using the fractions obtained from the gamma distribution model. Specifically, the fractions were considered for diffusion lower than 1 × 10^‐4^ mm^2^/s, and 3 grading thresholds were applied: Diffusion lower than 0.3 × 10^‐4^ mm^2^/s (ƒ11). Diffusion lower than 0.5 × 10^‐4^ mm^2^/s (ƒ12). Diffusion lower than 0.8 × 10^‐4^ mm^2^/s (ƒ13).

### 2.7. Statistical analysis

In this study, comprehensive and rigorous statistical analysis was performed to evaluate the data. First, the normality of the data was assessed using the Kolmogorov–Smirnov and Shapiro–Wilk tests to ensure that the assumptions of normality were met. Statistical analysis was carried out using IBM SPSS Statistics version 27.0.1 (×64), a widely used and powerful software in the field of data analysis. To compare different groups, the nonparametric Mann–Whitney U-test was employed, which is suitable for non-normally distributed data. This robustness test allowed us to determine the significant differences between the groups. Furthermore, to provide a comprehensive understanding of the model’s performance in differentiating between benign and malignant brain tumors, a receiver operating characteristic (ROC) analysis was utilized. The ROC analysis involved evaluating essential metrics, such as the area under the curve (AUC), sensitivity (%), specificity (%), and cutoff point values. The cutoff point values were computed by minimizing the differences between sensitivity and specificity, leading to optimal discrimination between the 2 groups. By employing these advanced statistical methods, we were able to thoroughly evaluate the data and gain valuable insights into the discriminative capability of the proposed model. Statistical analysis played a critical role in interpreting the results and drawing meaningful conclusions from this study.

## 3. Results

### 3.1. Staging

In the staging phase, a total of 73 patients (23–70 years old) participated, but 7 patients were excluded from the analysis due to poor quality of MRI images. Among the remaining participants, 24 were diagnosed with benign meningioma, comprising 10 females and 14 males. In addition, 42 patients were diagnosed with gliomas of different grades, including 16 females and 26 males. Regarding the grading phase, 18 of the glioma patients were classified as low-grade (grades I and II). Specifically, 9 patients had low-grade glioma, one had large B-cell lymphoma, 4 had astrocytoma (WHO Grade II), and 4 had choroid plexus papilloma (WHO Grade I). On the other hand, 24 patients were classified as having high-grade glioma, including 3 patients with glioblastoma (WHO Grade IV), 9 patients with glioblastoma, 2 patients with high-grade glioma with anaplastic astrocytoma, and 10 patients with anaplastic astrocytoma (WHO Grade III). These findings provide valuable insights into the distribution of different tumor types and grades among participants, establishing a strong foundation for further analysis and interpretation of the data.

The ADC and gamma distribution model parameters are listed in Table 1. As anticipated, the ADC values for benign tumors were higher than those for malignant tumors across all b values. Additionally, parameters κ, ƒ2, and ƒ3 were found to be higher in meningioma’s compared to gliomas, while parameter ƒ1 was higher in gliomas than in meningiomas.

**Table 1:** Statistical results for the gamma model parameters and ADC by different *b*_value in meningioma and glioma (staging step).

Parameters	*b* = 500(s/mm^2^)		*b* = 1000(s/mm^2^)		*b* = 1500(s/mm^2^)		*b* = 2000(s/mm^2^)	
	Meningioma	Glioma	Meningioma	Glioma	Meningioma	Glioma	Meningioma	Glioma
ADC (×10^‐4^ mm^2^/s)	6.2 ± 2.8	4.3 ± 2.6	7.4 ± 2.7	5.1 ± 2.6	6.3 ± 2.4	4.2 ± 2.5	6.2 ± 2.3	3.1 ± 3.0
Κ	.0503 ± .021	.0379 ± .019	.0575 ± .024	.0400 ± .04	.0558 ± .024	.0397 ± .022	.0623 ± .021	.0394 ± .038
Θ (*P* > .1)	74.55 ± 6.2	74.54 ± 5.1	74.55 ± 5.3	74.54 ± 5.4	74.55 ± 6.1	74.54 ± 4.8	74.55 ± 5.5	74.54 ± 6.1
ƒ_1_ (%)	82.79 ± 3.4	84.54 ± 3.06	81.15 ± 6.5	85.98 ± 2.8	83.39 ± 2.3	85.68 ± 1.9	82.38 ± 6.1	87.91 ± 3.5
ƒ_2_ (%)	8.25 ± 1.8	7.16 ± 1.8	9.39 ± .9	6.81 ± 1.7	10.21 ± 1.9	7.73 ± 1.4	10.79 ± 2.5	8.72 ± .9
ƒ_3_ (%)	9.15 ± 1.7	8.19 ± 1.2	6.4 ± 2.1	6.56 ± 1.9	6.37 ± 0.9	5.03 ± 1.2	6.34 ± 1.3	4.26 ± 1.2

ROC curve analysis was performed to further assess the performance of the model in differentiating between gliomas and meningiomas. The results, shown in Table 2, revealed that at *b* value of 1500 and 2000, the ADC values were significantly lower in gliomas than in meningiomas (*P* < .001), and the κ parameter was also significantly lower in gliomas with b value of 2000 (*P* < .001). Moreover, at b value of 1500 and 2000, fraction ƒ1 was significantly higher in gliomas (*P* < .001), while fraction ƒ2 showed significantly higher values in meningiomas (*P* < .001) at b value of 1500 and 2000. Furthermore, fraction ƒ3 exhibited higher values in meningiomas at b value of 1500 and 2000 (*P* < .001).

**Table 2:** ROC curve analysis of gamma distribution model parameters with different b values for meningioma and glioma.

ROC curve analysis		AUC	Standard error	*P*-value	Cutoff value	Sensitivity (%)	Specificity (%)
*b*_value, 500 s/mm^2^	ADC	0.728	0.063	.002	0.000470143	70%	62%
	κ	0.672	0.068	.02	0.0412	62%	63%
	ƒ_1_	0.733	0.072	.02	82.9	75%	63%
	ƒ_2_	0.671	0.071	.002	8.19	63%	63%
	ƒ_3_	0.699	0.074	.007	8.21	75%	61%
*b*_value, 1000 s/mm^2^	ADC	0.706	0.065	.004	0.000523615	69%	62%
	κ	0.724	0.074	.003	0.537	72%	77%
	ƒ_1_	0.817	0.073	.001	83.11	79%	72%
	ƒ_2_	0.750	0.037	.001	8.24	79%	63%
	ƒ_3_	0.720	0.069	.003	7.3	62%	56%
*b*_value, 1500 s/mm^2^	ADC	0.782	0.066	<.001	0.000469	75%	82%
	κ	0.713	0.065	.004	0.046	66%	70%
	ƒ_1_	0.864	0.063	.002	83.96	83%	68%
	ƒ_2_	0.777	0.043	<.001	8.76	88%	67%
	ƒ_3_	0.810	0.054	<.001	5.96	75%	83%
*b*_value, 2000 s/mm^2^	ADC	0.876	0.046	<.001	0.000362	75%	73%
	κ	0.845	0.051	<.001	0.0504	75%	89%
	ƒ_1_	0.895	0.059	<.001	85.81	95%	70%
	ƒ_2_	0.812	0.056	<.001	9.14	89%	76%
	ƒ_3_	0.891	0.042	<.001	4.85	83%	80%

The AUC values obtained from ROC curve analysis provided insights into the discriminative power of the model. At a *b*-value of 2000, AUC values of 0.895, 0.812, 0.891, 0.876, and 0.845 were achieved for ƒ1, ƒ2, ADC, and κ, respectively, for differentiating gliomas from meningiomas. At a *b*-value of 2000, the AUC was 0.891 for fraction ƒ3. Notably, the Ɵ parameter did not show significant differences across all b value and had consistent values (*P* > .1).

### 3.2. Grading

The results of the grading analysis considering different *b* values are presented in Table 3. For all *b* value, fractions ƒ11, were found to be higher in low-grade glioma, while parameters κ and ADC, were ƒ12, and ƒ13 higher in high-grade glioma.

**Table 3:** Statistical analysis of gamma distribution model and ADC with different *b*_value to differentiate between high-grade and low-grade glioma.

Types fractions & parameters	*b* = 500		*b* = 1000		*b* = 1500		*b* = 2000	
	Low grade	High grade	Low grade	High grade	Low grade	High grade	Low grade	High grade
ADC (×10^-4^ mm^2^/s)	3.7 ± 2.8	5.2 ± 3.2	1.3 ± 4.7	5.3 ± 2.4	3.1 ± 2.8	5.4 ± 2.9	2.3 ± 3.0	4.2 ± 3.1
κ	0.010 ± 0.009	0.018 ± 0.01	0.03 ± 0.01	0.04 ± 0.02	0.03 ± 0.03	0.06 ± 0.04	0.04 ± 0.05	0.08 ± 0.05
ƒ_11_ (%)	89.43 ± 0.8	87.98 ± 1.4	88.62 ± 0.9	86.56 ± 1.05	88.0 ± 1.4	85.0 ± 1.6	87.61 ± 2.3	84.76 ± 2.3
ƒ_12_ (%)	93.57 ± 0.5	94.03 ± 0.4	93.09 ± 0.7	93.81 ± 0.06	92.16 ± 1.3	93.68 ± 1.04	91.56 ± 1.9	93.4 ± 1.6
ƒ_13_ (%)	98.13 ± 0.5	97.71 ± 0.6	97.35 ± 0.7	98.01 ± 0.6	97.14 ± 0.7	98.01 ± 0.4	96.79 ± 1.04	98.91 ± 0.7

The ROC curve analysis, summarized in Table 4, demonstrated the effectiveness of using *b* value 1500 and 2000 in distinguishing between high-grade and low-grade tumors based on fractions ƒ11 < 0.3, ƒ12 < 0.5, and ƒ13 < 0.8 (*P* < .001). Additionally, in the ROC analysis using *b*-value 1500, the AUC was 0.840 for ƒ11 < 0.3 (*P* < .001) and 0.825 for ƒ13 < 0.8 (*P* < .001). Moreover, the AUC was significantly higher at *b*-value 2000 for ƒ12 < 0.5 (0.870), *P* < .001.

**Table 4:** Roc curve analysis of gamma distribution model parameters with different values of *b* to detect high-grade and low-grade tumors.

ROC curve analysis		AUC	Standard error	*P*-value	Cutoff value	Sensitivity (%)	Specificity (%)
*b*_value, 500 s/mm^2^	ADC (×10^‐4^ mm^2^/s)	0.685	0.08	.04	0.425	61%	68%
	κ	0.682	0.08	.04	0.053	89%	46%
	**ƒ**_11_ (%)	0.738	0.08	.009	89.16%	75%	67%
	**ƒ**_12_ (%)	0.729	0.07	.01	93.85%	62%	67%
	**ƒ**_13_ (%)	0.712	0.08	.02	98.03%	66%	61%
*b*_value, 1000 s/mm^2^	ADC (×10^-4^mm^2^/s)	0.713	0.08	.02	0.4018	67%	75%
	κ	0.727	0.08	.01	0.0339	67%	63%
	**ƒ**_11_ (%)	0.780	0.07	.002	88.39%	70%	78%
	**ƒ**_12_ (%)	0.748	0.08	.007	93.38%	83%	63%
	**ƒ**_13_ (%)	0.745	0.07	.007	97.60%	75%	50%
*b*_value, 1500 s/mm^2^	ADC (×10^-4^mm^2^/s)	0.727	0.08	.01	0.3541	72%	83%
	κ	0.786	0.07	.002	0.0343	72%	67%
	**ƒ**_11_ (%)	0.840	0.06	<.001	87.37%	83%	72%
	**ƒ**_12_ (%)	0.859	0.06	<.001	93.21%	87%	78%
	**ƒ**_13_ (%)	0.825	0.06	<.001	97.73%	72%	73%
*b*_value, 2000 s/mm^2^	ADC (×10^‐4^ mm^2^/s)	0.833	0.06	<.001	0.2138	83%	75%
	κ	0.829	0.06	<.001	0.0427	78%	75%
	**ƒ**_11_ (%)	0.815	0.06	<.001	87.19%	83%	73%
	**ƒ**_12_ (%)	0.870	0.06	<.001	93.17%	92%	72%
	**ƒ**_13_ (%)	0.812	0.06	<.001	97.73%	71%	73%

## 4. Discussion

The primary objective of this study was to assess the effectiveness of the gamma distribution model for grading and staging brain tumors. Our results demonstrated the model’s ability to distinguish between benign and malignant tumors. Furthermore, it exhibited a satisfactory performance in grading malignant tumors based on their malignancy levels. Our findings revealed a decrease in the average signal intensity with increasing *b*-values, accompanied by a reduction in the average κ and ADC parameters. It is noteworthy that meningioma tumors consistently displayed higher averages of ADC, κ, ƒ2, and ƒ3 across all b-value than glioma tumors. In line with Svolos et al’s findings, glioma tumors exhibited lower ADC values, with a stronger association between tumor cellularity and ADC, than meningioma tumors.^[9]^ These findings align with previous studies, such as You et al’s report, which also observed decreased signal intensity and average values of κ and ADC parameters with higher *b* values.^[2]^ Meyer et al similarly noted that ADC values can reflect tumor cellularity and microstructure, particularly in meningiomas and malignant tumors, where higher cellularity and larger nuclei lead to increased diffusion restriction and lower ADC values.^[10]^

The κ parameter serves as an index for assessing microstructural complexities in restricted water diffusion, indicating deviations from a normal distribution.^[11]^ In our study, the mean κ parameter values were consistently higher in meningiomas than in gliomas at all *b*-value (*P* < .001). This discrepancy may be attributed to the less restricted mobility of water molecules in meningioma tumors, resulting in higher ADC values and average κ values. Conversely, glioma tumors exhibit greater water molecule limitations, leading to lower ADC values and κ parameter averages.

The frequencies of ƒ1 and ƒ2 in gliomas and meningiomas vary according to the histological structure of the different tumors. Typically, gliomas have higher ƒ1 and lower ƒ2, whereas meningiomas exhibit lower ƒ1 and higher ƒ2. This distinction can be attributed to the benign nature of meningiomas, which are characterized by higher tissue cell density but rare necrotic areas.^[12]^ Reciprocally, glioma tumors not only display higher cell density than normal brain tissue but also contain necrotic regions with pronounced diffusion limitations. Our study corroborates these findings, indicating that, considering the κ and ADC parameters, ƒ1 is significantly higher in gliomas (*P* < .001). Ƒ1 was found to be the most effective parameter for distinguishing between meningioma and glioma using *b* = 2000 s/mm^2^ for diagnostic purposes (*P* < .001).

In biological tissues, water diffusion is influenced by the ratio of extracellular to intracellular space, reflected by ƒ2. Owing to increased extracellular diffusion, meningiomas tend to have higher ƒ2 values. Conversely, gliomas primarily encompassed hypercellularity areas, resulting in reduced extracellular space and lower water diffusion (*P* < .001). When evaluating tissue perfusion using the ƒ3 parameter (Frac > 0.3 × 10^‐4^ mm^2^/s), tumors resembling meningiomas exhibited superior tissue perfusion and consequently higher ƒ3 values. Contrarily, gliomas demonstrate reduced perfusion because of their tissue structure. Our study confirmed this hypothesis, with gliomas displaying lower ƒ3 values than meningiomas. As the *b* value increased from 0 to 2000 s to mm^2^, the differentiation between meningioma and glioma became more pronounced (*P* < .001), consistent with the findings of Huang et al, highlighting the highly vascular nature of meningiomas and their lack of a blood–brain barrier, resulting in enhanced perfusion compared to gliomas.^[13]^

The Ɵ parameter in the gamma distribution model can signify tissue heterogeneity.^[8]^ Although our hypothesis anticipated higher Ɵ parameter values in glioma tumors owing to their necrotic areas and increased heterogeneity compared to meningiomas, no significant difference was observed in utilizing this parameter to distinguish between the 2 tumor types. The values remained consistent across nearly all *b*-values (*P* > .1). This aligns with the findings of Tagao et al, indicating that the Ɵ parameter is ineffective in distinguishing between lymphomas and gliomas, potentially requiring a larger sample size for more conclusive results.^[8]^

**Figure 3.**
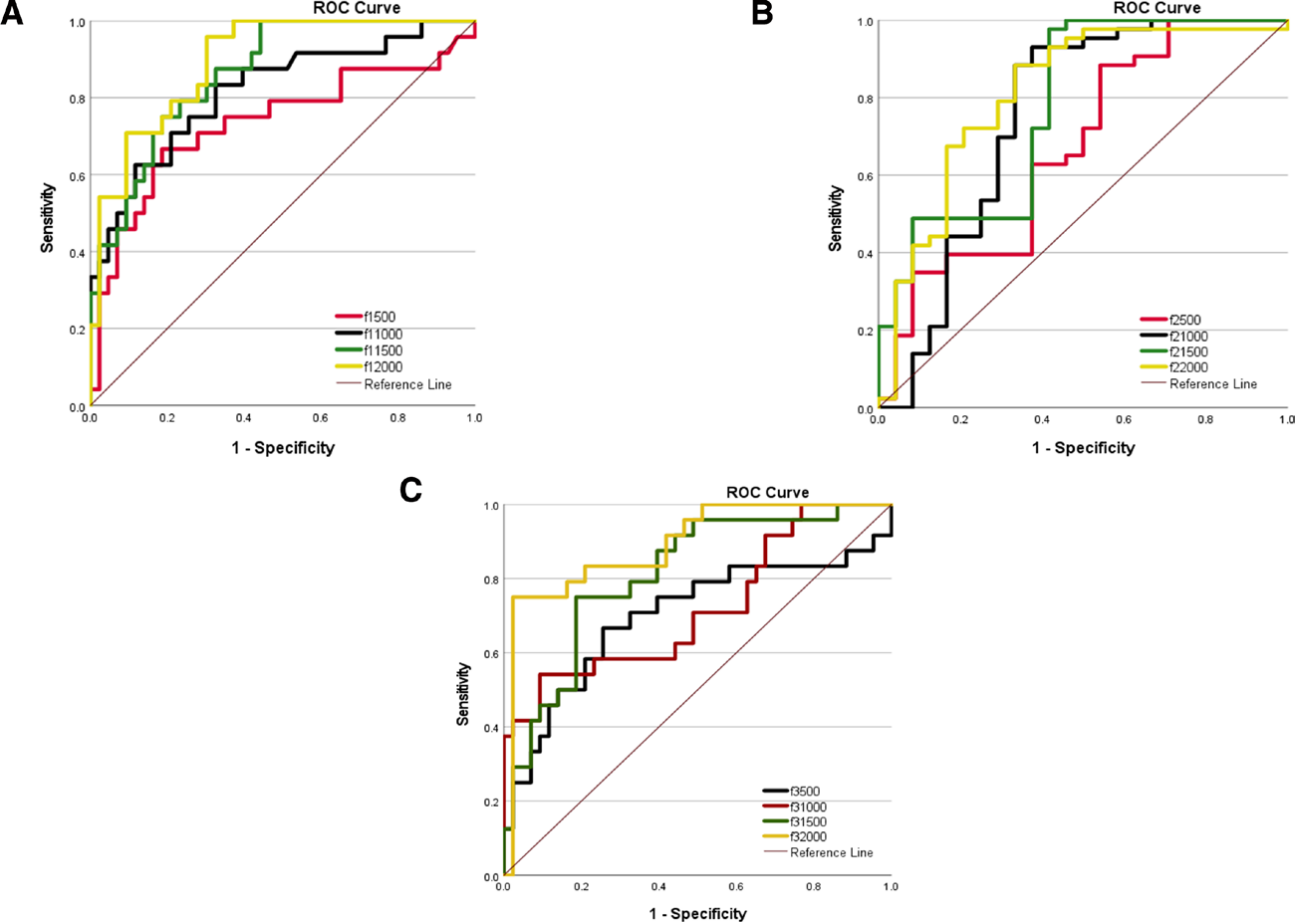
Receiver operating characteristic (ROC) curves for detection between meningioma and glioma with different *b* value. (A) ƒ_1_, (B) ƒ_2_, and (C) ƒ_3_.

As shown in Figure 3, when distinguishing between meningioma and glioma, ROC curve analysis revealed that the optimal performance was achieved at *b* = 2000 s/mm^2^. Both ADC and κ demonstrated comparable sensitivity at this level and effectively differentiated between the 2 tumor types. No preference was observed between ADC and κ. However, the ƒ1 fraction exhibited the best performance, boasting the highest sensitivity among all parameters. This underscores its reliability as a distinguishing parameter for meningioma and glioma. The second-best performance was attributed to ƒ2 and ƒ3, suggesting that these parameters, along with ƒ1, may provide more accurate differentiation between glioma and meningioma (*P* < .001).

**Figure 4.**
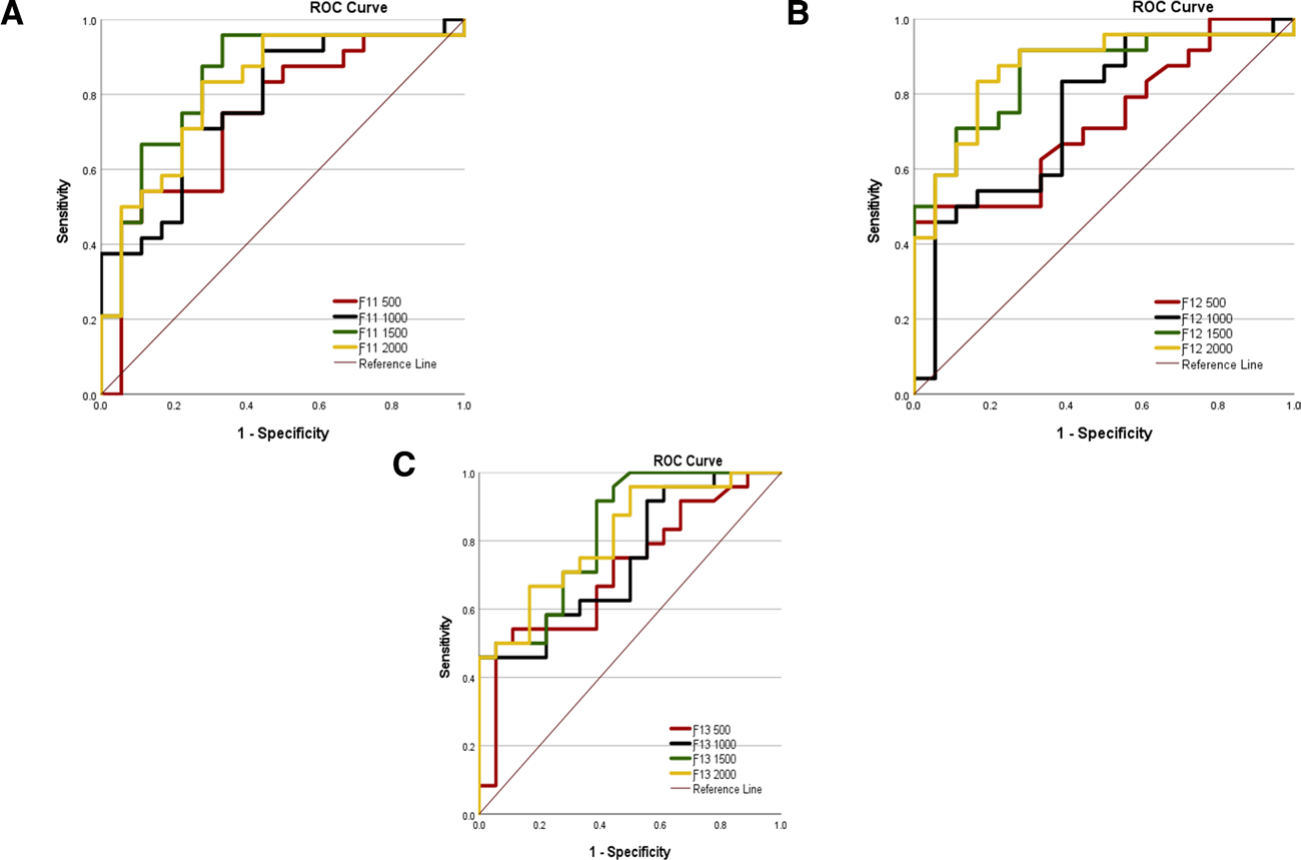
Receiver operating characteristic (ROC) curves for detection between meningioma and glioma with different *b*_value. (A) ƒ_11_, (B) ƒ_12_, (C) ƒ_13_.

In the context of glioma grading, it is essential to consider factors such as cellularity and vascularity, which significantly affect tumor characterization. High-grade gliomas are characterized by restricted diffusion areas on DWI images, primarily attributed to hypercellularity and prominent angiogenesis. Contrariwise, low-grade gliomas exhibit moderate hypercellularity and a slower growth rate, typically lacking necrotic regions, mitosis, and pronounced hypervascularity.^[14]^ In this phase of our study, we meticulously selected ROIs encompassing all enhancing lesions present on a single slice with the largest area on T1w post-contrast imaging. Our findings consistently demonstrated that high-grade gliomas exhibited notably higher ADC values than their low-grade counterparts did. This observation is consistent with the work of Sugahara et al, who reported elevated ADC values in high-grade gliomas relative to their low-grade counterparts.^[15]^ Server et al’s investigation sheds light on the potential of ADC as a differentiating factor, particularly in distinguishing metastatic tumors from high-grade gliomas. However, it is important to note that ADC faces challenges in distinguishing vasogenic edema induced by malignant tumors from tumor infiltration, as exemplified by conditions such as meningioma.^[16]^ Tropine et al underscored the impact of peritumoral edema and increased cellularity on ADC measurements, emphasizing the need for cautious interpretation when assessing these metrics.^[17]^ Our study extended these insights by revealing a consistent elevation in κ parameter within high-grade gliomas relative to their low-grade counterparts. The relationship between ADC values and tumor characteristics extends beyond gliomas and offers valuable insights into broader tumor biology. Togao et al’s research has highlighted the critical influence of the extracellular space on ADC measurements. Specifically, their study revealed that lymphomas characterized by a smaller extracellular space tend to exhibit lower ADC values due to a higher nucleus-to-cytoplasm ratio. In contrast, gliomas, characterized by prominent vasogenic edema, manifest higher ADC values, attributable to a larger extracellular space.^[8]^ The κ parameter, which is recognized as a reliable indicator of tissue microstructure, is notably influenced by the presence of peritumoral edema surrounding the tumor.^[8]^ Given the pronounced edematous regions associated with high-grade gliomas, the observation of higher κ parameter values in this context aligns with our expectation. Additionally, our investigation revealed a distinctive pattern wherein high-grade gliomas consistently displayed lower ƒ11 values across all b values compared to low-grade gliomas (*P* < .001). This finding is of particular significance as it underscores the potential diagnostic utility of ƒ11. Interestingly, we found that ƒ11, particularly when evaluated at *b* = 1500 s/mm², exhibited superior diagnostic performance compared with conventional ADC in distinguishing between high-grade and low-grade gliomas. In addition, in our study, a clear distinction emerged between high- and low-grade gliomas. High-grade glioma tumors consistently exhibited significantly larger values for both ƒ12 and ƒ13 compared to their low-grade counterparts (*P* < .001). These findings align seamlessly with prior research, providing further validation for the utility of these parameters in glioma characterization.^[8]^ Among the evaluated parameters, ƒ12 emerged as the most promising diagnostic indicator. Its diagnostic power surpassed that of ADC and κ, as evidenced by the consistently higher AUC across all examined *b* values. This indicates ƒ12’s potential to serve as a reliable discriminator between high- and low-grade gliomas. However, it is noteworthy that at a higher b value, no statistically significant diagnostic disparity was observed between ADC and κ. This was substantiated by the comparable AUC values at *b* = 2000 s/mm². In contrast, ƒ12 demonstrated exceptional diagnostic potential at this specific *b*-value, with the highest AUC among all the examined parameters. Additionally, ƒ13 exhibited its highest diagnostic efficacy at *b* = 1500 s/mm² compared to other *b* value (Fig. 4).

These observations collectively emphasize the nuanced interplay between diffusion-related parameters and their diagnostic utility for differentiating glioma grades. While ƒ12 shows remarkable promise, it is important to acknowledge the complementary roles that ADC, κ, ƒ11, and ƒ13 play in providing a comprehensive assessment of glioma characteristics.

However, our study, although informative, is not without limitations that warrant consideration. First, our patient selection criteria focused on individuals who had not undergone any prior diagnostic or treatment interventions for brain tumors. This stringent criterion, while ensuring a specific patient population, limits access to a larger and potentially more diverse cohort. Future studies may benefit from broader patient inclusion criteria to enhance the generalizability of the findings. Second, the inability to repeat the imaging protocol in cases of errors or suboptimal image quality introduced inherent variability into our data collection process. This limitation may have influenced the precision and reliability of our results. In future studies, implementing mechanisms for protocol repetition or refining imaging procedures could mitigate this issue. Third, we did not explore normal brain tissue characteristics or fit the normal tissue data to the gamma distribution model. Incorporating an analysis of normal brain tissue and comparing it with data derived from benign and malignant brain tumors could provide valuable insights into the distinctive features of tumor tissues. Future investigations should consider this aspect to enhance our understanding of brain tumor imaging. Moreover, the relatively low *b* value used in our MRI protocol represents another limitation. Increasing the range of *b* values in future studies could lead to more accurate and nuanced results and further enhance the diagnostic potential of diffusion-based parameters. Finally, our study employed an MRI scanner with a lower magnetic field strength. The utilization of a 3T MRI or a higher-powered scanner could offer more accurate and comprehensive imaging, potentially improving the study’s overall robustness and the quality of the obtained data.

## 5. Conclusion

The findings of our study highlight the potential of using a gamma distribution model with DWI based on multiple *b*-values for grading and staging brain tumors. Its potential integration into routine clinical practice could advance neurooncology and improve patient outcomes through more accurate diagnosis and treatment planning.

## Acknowledgments

We are very grateful to the staff of the imaging department of Isfahan Milad Hospital.

## Author contributions

**Conceptualization:** Zeinab Soleimani, Iraj Abedi.

**Data curation:** Zeinab Soleimani.

**Formal analysis:** Zeinab Soleimani.

**Investigation:** Iraj Abedi, Masih Saboori.

**Methodology:** Iraj Abedi.

**Project administration:** Iraj Abedi.

**Resources:** Zeinab Soleimani, Masih Saboori, Saeid Khanbabapour.

**Software:** Maziar Irannejad.

**Supervision:** Masih Saboori, Saeid Khanbabapour.

**Writing – original draft:** Zeinab Soleimani, Iraj Abedi.

**Writing – review & editing:** Iraj Abedi.
